# Contradictory Effects on Hepatocytes in ASMD

**DOI:** 10.3390/ijms27115070

**Published:** 2026-06-03

**Authors:** Maksim Sysoev, Dmitri Solovyov, Aleksandr Shestopalov, Sergey Kutsev

**Affiliations:** 1 Research Centre for Medical Genetics, 1 Moskvorechye St., 115522 Moscow, Russia; soldmtrlx@gmail.com (D.S.); al-shest@yandex.ru (A.S.);; 2 Department of Biochemistry and Molecular Biology, Institute of Pharmacy and Medicinal Chemistry, Pirogov Russian National Research Medical University, 1 Ostrovityanova St., 117997 Moscow, Russia

**Keywords:** hepatocytes, acid sphingomyelinase deficiency, acid sphingomyelinase, sphingomyelin, liver dysfunction, autophagy, lysosomes, lysosomal storage disease

## Abstract

Acid sphingomyelinase deficiency is a lysosomal storage disease that is characterized by the systemic accumulation of sphingomyelin in cells. This condition is frequently associated with hepatomegaly and hepatic dysfunction, with 91.4% of patients showing clinically relevant signs of liver involvement. Both clinical observations and experimental models show excessive sphingomyelin accumulation in hepatocytes. Studies using ASMD models have yielded conflicting results, showing hepatoprotective effects on one hand and detrimental effects on the other. Murine models demonstrated hepatoprotective effects of ASMD due to the modulation of endoplasmic reticulum stress. Patients with ASMD exhibit signs of impaired autophagy, which can lead to the accumulation of damaged cellular components and metabolic dysfunction. Furthermore, patients exhibit disrupted lipid metabolism, highlighting the dysfunction of hepatic lipid homeostasis. This review explores the involvement of ASMD in hepatocytes to better understand the disease mechanisms and possible therapeutic approaches.

## 1. Introduction

Acid sphingomyelinase deficiency (ASMD), encompassing Niemann–Pick disease types A, B, and A/B [1,2], is an autosomal recessive lysosomal storage disease caused by mutations in the *SMPD1* gene that results in the accumulation of sphingomyelin in various tissues [3,4]. Belonging to the sphingolipidoses subgroup of lysosomal storage diseases, ASMD has a birth prevalence of about 0.25–0.6 per 100,000 births, varying across countries and ethnic groups. Middle Eastern (8.04 per 100,000) and Ashkenazi Jewish (7.8 per 100,000) populations tend to have a higher prevalence of ASMD [5,6,7,8,9,10,11]. ASMD places a substantial burden on healthcare systems around the world [3,12,13,14,15,16,17].

In clinical classification, ASMD is divided into three types based on its severity and neurological involvement (Table 1). Patients with Niemann–Pick disease type A experience early onset and severe progression of the disease. They rarely have a lifespan longer than 2–3 years. In addition to significant hepatosplenomegaly in infancy, they suffer from severe and early-onset deterioration of the central nervous system [17,18,19]. Patients with type B also suffer from hepatosplenomegaly, liver and lung pathology, but show little to no neurological involvement. The age of onset and rate of disease progression vary greatly among patients with Niemann–Pick disease type B, and these patients often have a significantly longer life expectancy, living well into adulthood [20]. Finally, a third type, Niemann–Pick disease type A/B, has also been reported, characterized by intermediate levels of severity and pathological involvement of the CNS [21,22,23].

Hepatomegaly and liver dysfunction occur in 91.4% and 82.6% of patients, respectively, and liver failure is the most common cause of mortality in ASMD patients, accounting for 27.7% of deaths [26]. ASMD is an underdiagnosed and often treatable cause of hepatomegaly, splenomegaly, and low HDL cholesterol in lean individuals [27]. Patients with ASMD also have an elevated risk of bruising and bleeding, likely caused by thrombocytopenia and platelet dysfunction resulting directly from sphingomyelinase deficiency [13,28,29]. Variceal bleeding, resulting from the combination of liver failure and hemostatic abnormalities, is also a serious complication and a significant cause of death [26,30,31,32,33].

One of the unique pathological signs of ASMD is the formation of foamy macrophages [2,18,34,35,36,37]. A key role in the pathogenesis of ASMD has traditionally been attributed to the foamy macrophage infiltration in the liver, spleen, bone marrow and lungs of patients [34,35]. However, evidence is emerging that intrinsic functional mechanisms of target cells also play an important role.

## 2. Evidence of Hepatocyte Involvement in Patients

Liver, being the hub for many crucial biochemical pathways in the organism, is often one of the most affected organs in various metabolic disorders, including lysosomal storage diseases, such as ASMD [38]. Given that the liver also harbors a sizeable population of tissue-resident macrophages known as Kupffer cells, which are also susceptible to intracellular lipid accumulation due to their phagocytic function, the question arises whether intrinsic hepatocyte dysfunction is primary or secondary to macrophage dysfunction [38,39]. In most cases, excessive accumulation of sphingomyelin can be observed in hepatocytes [36,40]. However, in some patients, intracellular sphingomyelin accumulation in hepatocytes is not always found upon histological examination, and it is more often observed in patients with more severe cases of ASMD [40,41]. Liver organoids derived from a liver biopsy of a patient with ASMD also showed accumulation of lipids in the cytoplasm of hepatocytes [42].

**Figure 1:**
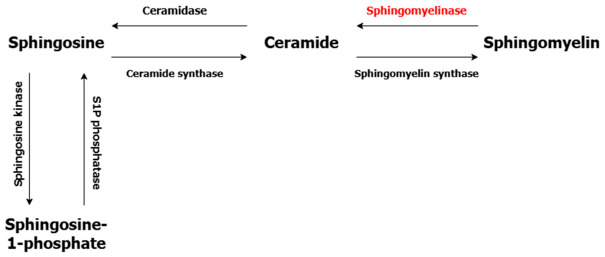
Schematic representation of the main sphingomyelin metabolism pathways.

Liver organoids derived from a patient with ASMD showed significantly higher expression of the gene *SMPD2*, which could be a compensatory mechanism to *SMPD1* deficiency. Interestingly enough, *SGMS1* expression was also elevated, which may seem contradictory at first glance, since *SGMS1* encodes sphingomyelin synthase 1, an enzyme expected to produce even more sphingomyelin (Figure 1) [42]. However, sphingomyelin synthase 1 is also able to catalyze a reverse reaction, converting sphingomyelin back into ceramide [5]. Considering the law of mass action, the reverse reaction happens under circumstances when there is excessive sphingomyelin. Thus, elevation of *SGMS1* expression may also be compensatory. Nonetheless, since the mechanisms causing this elevation remain unknown, further investigation is required. Among lipid metabolites besides sphingomyelin, triacylglycerol, cholesterol esters, lyso-phosphatidylethanolamine ether, and, surprisingly, ceramide were elevated, while phosphatidylcholine, diacylglycerol and cardiolipin were significantly decreased [42]. The paradoxical elevation of ceramide in spleen [43,44] and liver [45] tissues has also been observed in another sphingolipidosis, Gaucher disease, most likely through compensatory ceramide synthesis pathways [46]. The elevation of ceramide, though, is largely observed at the tissue or organ level, and differences in its cellular compartmentalization, as well as other lipids, in ASMD should be investigated [47,48,49].

Lipidomic profiling of liver organoids also revealed contradictory trends (Table 2). Ceramide is known to be a pro-inflammatory signaling molecule [50]. Triacylglycerol is usually considered to be pro-inflammatory when it contains long-chain saturated fatty acids, but can also exert anti-inflammatory effects, acting synergistically with omega-3 fatty acids when it contains medium-chain fatty acids [51,52]. Phosphatidylcholine is considered to have anti-inflammatory effects [53]. Diacylglycerol seems to have both anti- and pro-inflammatory effects, with anti-inflammatory effects more prominent in extracellular diacylglycerol, and pro-inflammatory effects supposedly more prominent in intracellular diacylglycerol [54,55,56]. Oxidized cardiolipin exerts pro-inflammatory effects, but native cardiolipin does not, and even has an anti-inflammatory effect via inhibition of caspase-4/11 in the non-canonical inflammasome pathway [57,58]. Lyso-phosphatidylethanolamine ethers usually exert anti-inflammatory effects, particularly when containing unsaturated acyl groups [59,60,61]. Sphingomyelin has both pro- and anti-inflammatory properties [62,63,64]. The buildup of cholesterol esters in the cell causes foam cell formation, contributing to a pro-inflammatory state [65,66]. Whether the recently discovered lipid lyso-SM-509 (PPCS)—which will be explored later in the review—is pro- or anti-inflammatory has not yet been determined [67,68].

A recent review demonstrated that dyslipidemia is a common manifestation of ASMD [72]. The majority of data shows that elevated plasma sphingomyelin levels are a risk factor for coronary artery disease [73,74]; however, some research yields conflicting results, suggesting the link between sphingomyelin and CAD may be complicated [75]. Patients with ASMD have a higher risk of cardiovascular involvement, and a murine model of ASMD has also shown significant cardiovascular dysfunction [12,76,77]. Several pathological mechanisms have been proposed. First, sphingomyelin accumulation in high-density lipoproteins disrupts biogenesis and maturation mediated by LCAT of newly formed high-density lipoproteins [78,79]. Additionally, sphingomyelin accumulation in HDL lowers additional SR-BI-mediated lipid influx from peripheral cells [80]. Moreover, sphingomyelin accumulation in macrophages causes defects in cholesterol transport and excretion [81]. Therefore, it is proposed that changes in lipid composition of HDL affect HDL catabolism, enhancing the faster removal of HDL from the circulation. Regarding LDL metabolism, it is hypothesized that excessive excretion of LDL is caused by increased bioavailability of lipids in the liver [82]. Meanwhile, clinical evidence suggests that slower removal of apoB-containing lipoproteins from circulation may be caused by high levels of PSCK9, a protein responsible for LDLR utilization [83]. Sphingomyelin also seems to cause the slowdown of the removal of apoE-containing lipoproteins from the circulation [84]. Furthermore, sphingomyelin appears to be a mixed inhibitor of lipoprotein lipase (LPL), which may contribute to atherogenic lipid profiles in patients [85]. Finally, there is a delay in chylomicron removal from the circulation [83]. The diversity of dyslipidemic effects likely stems from sphingomyelin having multiple targets, subsequently contributing to the proatherogenic lipid profile seen in patients with ASMD. A more in-depth investigation is needed to fully clarify these mechanisms.

Acid sphingomyelinase is an important regulatory protein in cell signaling. ASM activates PKB through the SphK/S1P pathway, decreases AMPK phosphorylation through ceramides, and increases GLUT2 expression in hepatocytes [86].

Therefore, evidence suggests that hepatocytes are likely to be directly involved in the pathogenesis of ASMD, rather than simply experiencing a secondary effect of macrophage infiltration in the liver [27,40,42].

## 3. Hepatoprotective Effects of ASMD in Models

ASM is essential for the normal functioning of hepatocytes; however, there is evidence that its inhibition may have a positive effect as well. It has been proposed that the acid sphingomyelinase–ceramide system could be a target involved in multiple pathways, which could be inhibited for the prevention of the progression of steatohepatitis [87]. In a murine model, it was shown that knockout of the acid sphingomyelinase gene protects mice against hepatic steatosis induced by a high-fat diet and methionine-choline-deficient diet; it was also shown that acid sphingomyelinase inhibition with amitriptyline, which is a functional inhibitor of acid sphingomyelinase (FIASMA) [88,89], prevents the development of non-alcoholic steatohepatitis induced by a high-fat diet [90]. Another study demonstrated that sphingomyelinase knockout mice were resistant to endoplasmic reticulum stress mediated by a high-fat diet and alcohol intake [90,91]. Moreover, lysosomal rupture caused by O-methyl-serine dodecylamide hydrochloride was blocked in ASM-deficient mice, but this protection was abolished by the depletion of lysosomal cholesterol in these mice, suggesting that lysosomal cholesterol might play a protective role in hepatocytes. The same was observed in palmitic acid-mediated lipotoxicity [90].

In another experiment using the murine model, acid sphingomyelinase deficiency blocked TNF-α-mediated apoptosis and necrosis of hepatocytes after mitochondrial glutathione depletion, protecting the liver from damage [92]. It has also been shown that ASM is important in hypoxia-induced apoptosis. ASM is activated under hypoxic conditions. Ceramide production leads to caspase 5 activation, which in turn cleaves ELAV-like protein 1, leading to hepatocyte apoptosis [93]. Pharmacologically induced ASM inhibition with amitriptyline and genetic knockout of *SMPD1* protected liver cells from ethanol-mediated cytotoxicity and ER stress [91]. ASM knockout also reduced mitochondrial cholesterol accumulation in hepatocytes induced by ethanol [91].

Notably, acid sphingomyelinase controls hepatic stellate cell activation and in vivo liver fibrogenesis, which affects the function of hepatocytes and contributes to ASMD pathology [94].

Inhibition of ASM lowers the occurrence of sepsis-mediated liver damage. In a murine model, it was shown that heterozygous *SMPD1* knockout and pharmacological inhibition of ASM with desipramine, which is a FIASMA [89,95], notably improved post-sepsis survival and liver function by reducing hepatic stellate cell activation [96]. Acid sphingomyelinase levels in plasma also correlate with sepsis severity and can be used as a prognostic marker for the outcome of sepsis [97].

## 4. The Role of Acid Sphingomyelinase in Lysosomal Repair

Lysosomes are crucial organelles in hepatocytes, playing critical roles in lipid metabolism and homeostasis as well as autophagy [98]. Permeabilization of the lysosomal membrane leads to the leakage of potent hydrolytic enzymes into the cytoplasm, among which cathepsins are established mediators of several different cell death pathways [99,100,101]. Thus, repairing damaged lysosomal membranes is essential for maintenance of cellular homeostasis and prevention of lysosomal cell death [100].

ESCRT-mediated repair and phosphoinositide-initiated membrane tethering and lipid transport (PITT) are considered to be the main lysosomal repair pathways; however, there are alternative pathways that involve sphingomyelin [100,102,103]. Damage to lysosomal membranes causes the Ca^2+^-dependent relocalization of sphingomyelin from the internal to the external side of the lysosomal membrane [103,104,105]. In one mechanism, the protein TECPR1 is relocated to the site of lysosomal damage by means of its dysferlin domain, which is specific to membrane sphingomyelin [106,107,108]. Once relocated, TECPR1 forms a complex with ATG12-ATG5 proteins, which amplifies the lipidation of LC3B protein in the damaged sites via an alternative pathway that does not depend on ATG16L1 [107,108]. Consequently, this implies that accumulation of sphingomyelin is supposed to enhance lysosomal repair, but research has shown that excessive accumulation of sphingomyelin causes damage to lysosomal membranes and autophagy disruption in ASMD type A [109].

Another mechanism includes the action of acid sphingomyelinase and biophysical properties of ceramide: relocalized to the external membrane of lysosomes, sphingomyelin is hydrolyzed into ceramide by ASM [104]. Since ceramide occupies a smaller surface area than sphingomyelin and has a conical geometric structure, it curves the membrane inward, which contributes to the repair of the damaged lysosomal membrane [100,110]. In vitro controlled lysosomal damage induced by LLOMe demonstrated that lysosomal sphingomyelin decreases and ceramide increases significantly, which is in agreement with the mechanism [111]. During fusion of lysosomes with the membrane of the cell, when ceramide is generated, caveolae-mediated endocytosis is promoted [112,113]. The main ESCRT-mediated lysosomal repair pathway and ceramide generation may aid each other synergistically in optimal efficiency of lysosomal membrane repair [102].

Ceramide generation on the internal side of the lysosomal membrane, when it is not excessive [114], is also associated with higher lysosomal membrane stability [115]. The complex formed by heat shock protein 70 (Hsp70) and the endolysosomal lipid bis(monoacylglycero)phosphate (BMP) has been shown to stabilize lysosomal membranes by enhancing the activity of ASM [116,117]. Additionally, ASM-mediated hydrolysis of sphingomyelin to ceramide enhances the ordering of membrane acyl chains and promotes tighter lateral packing of lipids [118]. Furthermore, ceramide improves the ability of lysosomal membranes to fuse with the membranes of other organelles and the extracellular membrane [119,120]. However, ceramide has been determined to be elevated in a liver organoid model of ASMD, so the exact pathways of these mechanisms require further investigation in ASMD models and biological tissues of patients [42].

## 5. ASMD Effects on Autophagy in Hepatocytes

Autophagy is a lysosome-mediated intracellular degradation pathway that is essential for the maintenance of metabolic and energetic homeostasis, organelle quality control and regulation of intracellular signaling pathways [121,122,123]. Autophagy is especially crucial for liver function, partly because of its role in supplying free fatty acids for β-oxidation via lipophagy, and mobilization of glucose from glycogen via glycophagy [123,124]. The process starts with the engulfment of the target by a phagophore—an open double membrane structure that is assembled on a specialized PI3P-enriched domain of the endoplasmic reticulum called the omegasome [122,125]. The phagophore then expands and closes around the cargo, forming an autophagosome, which fuses with a lysosome for terminal enzymatic degradation of its contents [109,122,126].

Dysregulation of autophagy is commonly observed in various lipid storage diseases [127]. ASMD presents with the most severe liver involvement among the sphingolipidoses, and is characterized by the accumulation of sphingomyelin lipid in hepatocytes specifically [38]. In contrast, in Gaucher disease, hepatomegaly is attributed to infiltration by Gaucher cells resulting from the accumulation of glucosylceramide in macrophages rather than hepatocytes [38]. Liver failure is also significantly less common in Gaucher disease compared to ASMD [38], suggesting that hepatocytes are the primary targets of autophagy failure in ASMD.

Signs of impaired autophagy, such as accumulation of autophagosomes, ubiquitinated proteins and abnormal, elongated phagophores have been observed in various types of ASM-deficient human cells and murine models of Niemann–Pick type A [122,126,128]. In ASM-deficient mice, accumulation of SQSTM1 and LC3B-II in hepatocytes and other cell types was revealed, indicating an impairment in autophagic flux [122]. In another murine model, *SMPD1* knockdown in arterial smooth muscle cells led to a decrease in autophagolysosome formation due to disruption of autophagosome-lysosome fusion [126]. Intravenous administration of the mTOR-independent autophagy inducer trehalose leads to an improvement in clinical symptoms and serum sphingomyelin metabolite levels in patients with Niemann–Pick disease type A and B [121,129,130].

Autophagosome formation and maturation are tightly regulated by the ATG family of proteins [122,125]. In ASM-deficient cells, ATG-9 is concentrated in the juxtanuclear region of the Golgi apparatus and colocalizes with the transferrin receptor TfR1, which, after its activation, is trafficked to recycling endosomes in a sphingomyelin-dependent manner. Treatment of control fibroblasts with exogenous C12- and C16-sphingomyelin recaptures the changes in ATG-9 distribution observed in ASM-deficient cells, indicating that they are caused by the excessive levels of sphingomyelin, rather than a shortage of its derivatives [122].

Lysosomal storage disorders are associated with pathological changes in Ca^2+^ homeostasis, which might contribute to impairments in autophagy regulation [131]. TRPML-1 is an ion channel permeable to many ions, including Ca^2+^. Lysosomal calcium release through TRPML-1 contributes to autophagy regulation by promoting lysosome motility and fusion with autophagosomes and activating mTORC1 to inhibit autophagy [131,132]. Additionally, TRPML-1-mediated Ca^2+^ release leads to activation of serine/threonine protein phosphatase calcineurin, leading to dephosphorylation and subsequent nuclear translocation of TFEB—a major regulator of lysosomal biogenesis and autophagy [131,133]. Two-pore channels (TPCs) are another group of Ca^2+^ channels present on lysosomes; they are known to induce autophagy in a NAADP-dependent manner [131,134]. Importantly, it has been shown that sphingomyelin accumulation inhibits TRPML-1-mediated Ca^2+^ release, causing a significant lysosomal trafficking defect [135,136,137].

Ultimately, the ASM protein plays an important role in the regulation of autophagy, and its deficiency in cells causes significant impairment of autophagic function, which, among other factors, likely contributes to the pathology observed in ASMD (Table 3).

## 6. Hepatocellular Nitric Oxide Synthase Activity in ASMD

Nitric oxide synthases are a family of enzymes that catalyze nitric oxide synthesis from L-arginine [140,141]. Of the three NOS isoforms, expression of inducible NOS and endothelial NOS has been confirmed in human hepatocytes [138]. Constitutive isoforms of NOS produce NO under physiological conditions as a messenger molecule regulating vascular tone and serving as a neurotransmitter in the central nervous system [141]. In the immune response, NO, mainly produced by iNOS, and its highly reactive derivatives take part in the direct elimination of pathogens alongside reactive oxygen species [140]. Nitric oxide is also shown to play contradictory roles in the pathogenesis of various metabolic disorders, such as diabetes and obesity, through a variety of mechanisms [138,141]. In hepatocytes, it inhibits gluconeogenesis through cGMP-dependent mechanisms, and possibly, S-nitrosylation of relevant enzymes, playing a protective role in type 2 diabetes mellitus [138].

Expression of iNOS in hepatocytes is triggered in response to various inflammatory and otherwise damaging factors, such as Kupffer cell activation [142], reactive oxygen species production, ischemia–reperfusion injury, and exposure to endotoxins and proinflammatory cytokines [138]. Upregulation of iNOS by hepatocyte growth factor and oncostatin-M promotes hepatogenic differentiation of human MSCs by inhibiting the Notch pathway via S-nitrosylation of NOTCH1 [143].

In murine models, iNOS has been shown to promote nonalcoholic fatty liver disease and obesity via impaired autophagy in hepatocytes and macrophages [144,145], but also to play a protective role in liver ischemia–reperfusion injury [146,147]. In isolated murine hepatocytes, iNOS inhibition leads to an enhanced LDL clearance by promoting the expression of the LDL receptor and inhibiting its antagonist PCSK9 in a mTORC1-dependent manner [148].

There is emerging evidence of hepatocellular eNOS playing a role in NAFLD pathogenesis [149]. In murine models, eNOS knockout led to impaired mitochondrial function and turnover in hepatocytes, exacerbating NAFLD [150,151,152,153,154]. Sphingosine-1-phosphate and its receptors S1PR1 and S1PR3 regulate eNOS activity via the PKB pathway [155,156,157]. In diabetic mice, S1P restored S1PR1-3 expression in myocardial tissue and led to an increase in serum eNOS levels [158].

Nitric oxide inhibits ASM and downstream ceramide production in a cGMP-dependent manner. This has been shown to ameliorate the proapoptotic effects of E. coli-derived LPS in murine dendritic cells [159]. Ceramide accumulation promotes the colocalization of protein phosphatase 2A with eNOS and disrupts eNOS activation [160]. ASM downregulation recovered eNOS and NO levels in rat endotheliocytes with palmitate-induced insulin resistance, further confirming its ceramide-mediated inhibitory effect on eNOS and role in the development of diabetes [139].

## 7. Lysosphingomyelin-509

Lysosphingomyelin-509, known as lyso-SM-509 for short, or by its new name following structural identification, N-palmitoyl-O-phosphocholineserine (PPCS), is a lipid that is elevated in the tissues and plasma of patients with acid sphingomyelinase deficiency and Niemann–Pick disease type C (NPC). It is an emerging diagnostic biomarker for the screening of these disorders [67,69,70,71]. PPCS has been shown to be synthesized and to accumulate significantly in the liver in a cat model [161], suggesting that hepatocytes are key PPCS-producing cells. Even though its precise chemical structure has been determined, the mechanisms of its formation in the cell and its role in molecular physiology and pathophysiology of Niemann–Pick disease remain incompletely understood, although hypotheses about its synthesis and degradation have been proposed [67,68,162].

**Figure 2:**
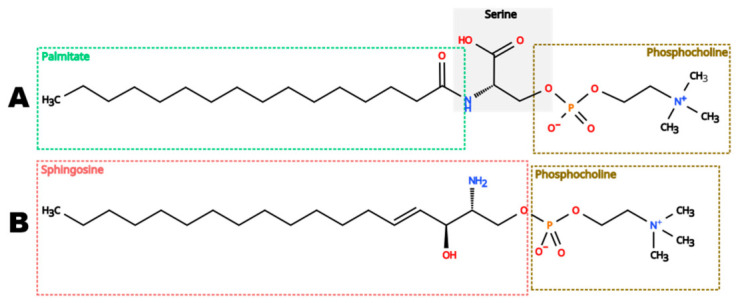
Chemical structures of N-Palmitoyl-O-phosphocholineserine (lyso-SM-509) (**A**) and lyso-sphingomyelin (**B**). Images borrowed from the National Center for Biotechnology Information. PubChem Compound Summary for CID 169445597 and 6437839, respectively, N-Palmitoyl-O-phosphocholineserine: https://pubchem.ncbi.nlm.nih.gov/compound/N-Palmitoyl-O-phosphocholine-Serine. Accessed 13 May 2026. Sphingosine phosphorylcholine: https://pubchem.ncbi.nlm.nih.gov/compound/Sphingosine-phosphorylcholine. Accessed 13 May 2026.

Most likely, lyso-SM-509 is not directly derived from sphingomyelin and is not truly related to lyso-SM apart from having phosphocholine in its structure (Figure 2). Thus, synthesis of lyso-SM-509 requires a yet unknown metabolic pathway. Interestingly, pathological variants of *APOE* have also been identified as a cause of elevated PPCS levels in blood serum, suggesting a link between lipoprotein metabolism dysfunction and PPCS accumulation [163]. Gaucher disease, which has hepatomegaly as a hallmark feature [164], and congenital disorders of glycosylation (CDG) with significant liver involvement, such as PMM2-, ALG1-, ALG8-, and ATP6AP1-CDG [165] also feature elevated serum lyso-SM-509 levels [166,167,168]. Remarkably, trehalose intake causes lyso-SM-509 to decrease in certain patients with ASMD, showing a link between the metabolite and impaired autophagy [129]. Its ligand-receptor interactions remain unknown and have yet to be identified [161,169].

## 8. Conclusions

The dual nature of ASMD’s impact on hepatocytes—exhibiting both protective and detrimental signatures—presents a relevant issue in lysosomal pathology. Several hypotheses may explain these paradoxical findings.

Sphingomyelin appears to be a multifunctional regulatory lipid molecule, serving as an inhibitor of some targets and as an activator of others. It is possible that the contradictory effects of sphingomyelinase inhibition in hepatocytes and the clinical manifestations of ASMD arise from the dual, context-dependent role of sphingomyelinase in regulating both cell survival/homeostasis and pro-inflammatory/apoptotic signaling, depending on whether it acts within the lysosome or at the plasma membrane.

One possible explanation lies in the concept of mitohormesis and adaptive stress responses. In early stages or specific murine models, the initial accumulation of sphingomyelin may trigger a robust, albeit transient, cytoprotective response. By slightly altering the properties of the endoplasmic reticulum (ER) membrane, ASM deficiency might protect the cell from acute ER-stress-induced apoptosis, as observed in some studies. However, as the disease progresses, the cells’ adaptive capacity becomes overwhelmed, leading to chronic metabolic failure [170,171]. In short, while absolute or chronic deficiency (as in ASMD) leads to massive sphingomyelin storage, partial or pharmacological inhibition of ASM can, in specific scenarios, provide protective effects against liver injury. Notably, pharmacological inhibition or partial genetic silencing can alleviate liver injury in models of sepsis, and these protective effects are possibly achieved by limiting excessive ceramide-induced inflammatory responses. This is because acute activation of ASM, rather than its basal activity, produces excessive ceramide and pro-inflammatory signaling, such as the NF-κB pathway that drives damage [172,173].

Inhibition prevents the conversion of sphingomyelin into pro-apoptotic and pro-inflammatory ceramide, and it can trigger autophagy due to alterations in lysosomal membrane lipid composition. These alterations are caused by an increase in sphingomyelin content, which may initially help in clearing damaged organelles before eventually leading to the storage crisis seen in full-blown ASMD. In addition, ASMD may cause even higher levels of ceramide than normal [42], as shown in this review, which worsens the inflammatory state of the patients.

The contradictory effects may also stem from the distinct roles of ASM in different cellular compartments [174,175]. The lysosomal accumulation of sphingomyelin clearly appears to be pathogenic. Excessive sphingomyelin accumulation causes the disruption of autophagosome-lysosome fusion through the disturbance of ATG9A trafficking [122]. Despite sphingomyelin being a key molecule in lysosomal repair [106,107,108], high sphingomyelin levels in lysosomes have actually been shown to block lysosomal repair. This likely happens through blockage of ceramide-mediated lysosomal repair described earlier in the text [100,110,111]. The buildup of sphingomyelin inhibits TRPML1-mediated Ca^2+^ signaling in lysosomes [135,136,137], also potentially affecting lysosomal membrane repair [176]. In contrast, a deficiency of secretory ASM may block the formation of ceramide-rich platforms on the cell membrane, decreasing pro-apoptotic and pro-inflammatory effects [177]. Similarly, low ASM activity may lead to protection from ER stress through blockage of ceramide generation [178,179]. The net effect on the hepatocyte likely depends on the balance between these processes [180].

The hepatoprotective effects observed in some animal models may not fully translate to human physiology due to specific differences in lipid metabolism and lifespan. Significant metabolic divergence exists between human and murine hepatic systems [181]. Moreover, in patients, the chronic, decades-long accumulation of lipids leads to secondary phenomena—such as impaired autophagy and defective HDL biogenesis—which are difficult to replicate in short-term animal experiments. Thus, the “protective” signature may result from the biological model’s timeframe or specific genetic background [182].

It is also possible that ASMD-affected hepatocytes are not inherently destined for death but rather exist in a state of “primed vulnerability”. In this scenario, sphingomyelin accumulation acts as a first hit that alters autophagy and lipid flux. The final detrimental outcome (fibrosis or cirrhosis) may require a “second hit”, such as systemic inflammation driven by foamy macrophages or oxidative stress, which converts a subclinical metabolic state into overt liver failure [183].

In conclusion, the perceived contradiction may be explained by multiple hypotheses, but several important questions remain unresolved and should be addressed in future studies. The development of hepatocyte-specific ASM conditional knockout models may help distinguish direct hepatocellular effects of ASM deficiency from secondary effects driven by macrophages, systemic inflammation, or other cell types such as hepatic stellate cells. The usage of genetically encoded biosensors such as lysenin or fluorescent SM analogs such as BODIPY-SM [184] for tracking sphingomyelin trafficking and distribution between lysosomes, the Golgi apparatus, secretory vesicles, and plasma membranes may clarify the compartment-specific functions of ASM and sphingomyelin. Finally, characterization of PPCS (lyso-SM-509), including the identification of its receptors, signaling pathways, and physiological functions, may provide important insights into ASMD pathogenesis and promote the development of novel biomarkers and therapeutic targets. Thus, further research is required to more precisely identify the mechanisms that govern the balance between the protective and harmful effects of ASMD on hepatocytes.

## Figures and Tables

**Table 1:** Comparison of ASMD clinical types.

ASMD Type	Severity	CNS Involvement	Life Expectancy	Key Clinical Manifestations
A	Most severe [18]	Present, very pronounced, rapid progression [18]	Greatly reduced (usually <2–3 years) [18]	Hepatosplenomegaly combined with rapid neurodegeneration [18]
B	Least severe [20]	Minimal to none [20]	Usually reduced, but live well into adulthood [20,24,25]	Hepatosplenomegaly, liver and lung pathology [20]
A/B	Variable severity [21,22]	Present, slow progression [21,22]	Reduced, depending on disease severity [21,22,24,25]	Hepatosplenomegaly, liver and lung pathology, slow neurodegeneration [21,22]

**Table 2:** Profile changes in lipids in ASMD and their respective proposed effects.

Lipid	Levels	Proposed Effect
Ceramide	Increased [42]	Pro-inflammatory [50]
Sphingomyelin	Increased [42]	Exhibits both pro- and anti-inflammatory effects [62,63,64]
Triacylglycerol	Increased [42]	Usually pro-inflammatory, can be anti-inflammatory under certain circumstances [51,52]
Cholesterol esters	Increased [42]	Pro-inflammatory (secondary effect) [65,66]
Lyso-phosphatidylethanolamine ether	Increased [42]	Anti-inflammatory [59,60,61]
Phosphatidylcholine	Decreased [42]	Anti-inflammatory [53]
Diacyglycerol	Decreased [42]	Intracellular localisation: usually pro-inflammatory Extracellular localisation: usually anti-inflammatory [54,55,56]
Cardiolipin	Decreased [42]	Oxidized form: pro-inflammatory Native form: anti-inflammatory [57,58]
Lysosphingomyelin-509 (N-palmitoyl-O-phosphocholine serine)	Increased [67,69,70,71]	Unknown [67,68]

**Table 3:** Effects of ASMD and ASM inhibition in patients and models.

Effects	Hepatocyte or Liver-Related Effects	Evidence
Negative	Liver failure and dysfunction	Hepatomegaly, cirrhosis, high mortality from liver failure [26]
	Impairment of autophagy	Accumulation of autophagosomes, ubiquitinated proteins, abnormal phagophores [122,126,128], disruption of lysosomal fusion [126]
	High atherogenic lipid profile	Multiple hypothetical targets, see in text [72]
	Defective lysosomal repair	Failure of ceramide generation on the external lysosomal membrane resulting in defective ASM-mediated alternative pathway of lysosomal repair [100,104,110]
Positive	Resistance to steatosis	ASM knock-out [88,89] and ASM-inhibited mice are resistant to fatty liver disease induced by a high-fat diet [90]
	Protection against cytotoxicity and ER stress	Inhibition of ASM in mice protects against ethanol-mediated cytotoxicity and ER stress [91]
	Block of apoptosis	ASMD blocks TNF-α-mediated apoptosis and necrosis in murine models [92]
	Hypothetically enhanced lysosomal repair (?)	Sphingomyelin causes SM-specific protein TECPR1 to form a complex with ATG12-ATG5 proteins, amplifying the lipidation of LC3B [107,108]
	Protection against insulin resistance	Sphingosine-1-phosphate promotes eNOS expression via S1PRs, leading to inhibition of gluconeogenesis and alleviation of insulin resistance [138,139]

## Data Availability

No new data were created or analyzed in this study.

## Abbreviations

The following abbreviations are used in this manuscript:

ASMD	Acid sphingomyelinase deficiency
HDL	High-density lipoprotein
CNS	Central nervous system
lyso-SM-509	Lysosphingomyelin-509
PPCS	N-Palmitoyl-O-phosphocholineserine
CAD	Coronary artery disease
LCAT	Lecithin-cholesterol acyltransferase
SR-BI	Scavenger receptor class B, type I
LDL	Low-density lipoprotein
apoB	Apolipoprotein B
PCSK9	Proprotein convertase subtilisin/kexin type 9
LDLR	Low-density lipoprotein receptor
apoE	Apolipoprotein E
LPL	Lipoprotein lipase
ASM	Acid sphingomyelinase
PKB	Protein kinase B
SphK	Sphingosine kinase
S1P	Sphingosine-1-phosphate
AMPK	Adenosine monophosphate-activated protein kinase
GLUT2	Glucose transporter 2
FIASMA	Functional inhibitors of acid sphingomyelinase
TNF-α	Tumor necrosis factor alpha
ELAV	Embryonic lethal abnormal vision
ER	Endoplasmic reticulum
ESCRT	Endosomal sorting complexes required for transport
PITT	Phosphoinositide-initiated membrane tethering and lipid transport
TECPR1	Tectonin beta-propeller repeat containing 1
ATG	Autophagy-related
LC3B	Microtubule-associated protein 1 light chain 3 beta
ATG16L1	Autophagy-related 16 like 1
LLOMe	L-Leucyl-L-Leucine methyl ester
Hsp70	Heat shock protein 70
BMP	Bis(monoacylglycero)phosphate
PI3P	Phosphatidylinositol 3-phosphate
SQSTM1	Sequestosome-1
LC3B-II	Lipidated, membrane-bound form of Microtubule-associated protein 1 light chain 3 beta
TRPML1	Transient receptor potential mucolipin 1
TfR1	Transferrin receptor 1
mTORC1	Mammalian target of rapamycin complex 1
TFEB	Transcription factor EB
TPC	Two-pore channels
NAADP	Nicotinic acid adenine dinucleotide phosphate
SM	Sphingomyelin
eNOS	Endothelial nitric oxide synthase
S1PR	Sphingosine-1-phosphate receptor
NOS	Nitric oxide synthase
iNOS	Inducible nitric oxide synthase
cGMP	Cyclic guanosine monophosphate
MSCs	Mesenchymal stem cells
NOTCH1	Neurogenic locus notch homolog protein 1
NAFLD	Non-alcoholic fatty liver disease
NO	Nitric oxide
NPC	Niemann–Pick disease type C
lyso-SM	Lysosphingomyelin
CDG	Congenital disorders of glycosylation
PMM2	Phosphomannomutase 2
ALG1	Chitobiosyldiphosphodolichol beta-mannosyltransferase
ALG8	Alpha-1,3-glucosyltransferase
ATP6AP1	Adenosine triphosphatase H+ transporting accessory protein 1
CID	Compound identification
NF-κB	Nuclear factor kappa-light-chain-enhancer of activated B cells
BODIPY	boron–dipyrromethene
